# Mucosal Administration of E-selectin Limits Disability in Models of Multiple Sclerosis

**DOI:** 10.3389/fnmol.2019.00190

**Published:** 2019-08-27

**Authors:** Jacqueline A. Quandt, Pierre Becquart, Emily Kamma, John Hallenbeck

**Affiliations:** ^1^Department of Pathology and Laboratory Medicine, University of British Columbia, Vancouver, BC, Canada; ^2^Stroke Branch, National Institute of Neurological Disorders and Stroke, National Institutes of Health, Bethesda, MD, United States

**Keywords:** multiple sclerosis, E-selectin, mucosal tolerance, experimental autoimmune encephalomyelitis, neuroinflammation

## Abstract

E-selectin plays an important role in mediating the rolling of leukocytes along and thus, the subsequent extravasation across activated endothelial cells comprising the microvasculature of the blood brain barrier (BBB). In multiple sclerosis (MS) and other inflammatory disorders of the central nervous system (CNS), the microvasculature is altered and immune cells infiltrate the brain and spinal cord contributing to damage, demyelination and ultimately disability. While mucosal administration is typically used to affect lymphocyte hyporesponsiveness or tolerance to suspect autoantigens, intranasal administration to E-selectin has previously been shown to protect against CNS inflammatory insults. We characterized the potential for mucosal administration of E-selectin to modulate CNS autoimmunity in the experimental autoimmune encephalomyelitis (EAE) model of MS. Intranasally administered E-selectin reduced swelling by as much as 50% in delayed-type hypersensitivity reactions compared to ovalbumin-tolerized controls. Intranasal E-selectin delivery prior to disease induction with myelin oligodendrocyte glycoprotein (MOG)_35–55_ reduced disease severity and total disease burden by more than 50% compared to PBS-tolerized animals; this protection was not associated with differences in the magnitude of the autoimmune response. Examination after the onset of disease showed that protection was associated with significant reductions in inflammatory infiltrates throughout the spinal cord. Tolerization to E-selectin did not influence encephalitogenic characteristics of autoreactive T cells such as IFN-gamma or IL-17 production. Clinical disease was also significantly reduced when E-selectin was first delivered after the onset of clinical symptoms. Splenic and lymph node (LN) populations from E-selectin-tolerized animals showed E-selectin-specific T cell responses and production of the immunomodulatory cytokine IL-10. Transfer of enriched CD4+ T cells from E-selectin tolerized mice limited disability in the passive SJL model of relapsing remitting MS. These results suggest a role for influencing E-selectin specific responses to limit neuroinflammation that warrants further exploration and characterization to better understand its potential to mitigate neurodegeneration in disorders such as MS.

## Introduction

Multiple sclerosis (MS) is amongst the most common neurological diseases in young adults, with symptoms such as altered balance, vision or speech, weakness or paralysis, as well as extreme fatigue, depression and cognitive dysfunction. MS is a chronic inflammatory neurodegenerative disorder characterized histologically by vascular changes, immune infiltrates, loss of myelin and oligodendrocytes, and damage or loss of axons (Frohman et al., 2006). Ultimately, chronic disability is closely linked to neurodegeneration and irreversible axonal loss (Bjartmar and Trapp, 2003). Oligodendrocytes, myelin, neurons and axons are all damaged (Frischer et al., 2009) in MS and its primary animal model, experimental autoimmune encephalomyelitis (EAE). The demyelination and axonal injury observed in acute inflammatory lesions (Trapp et al., 1998; Kuhlmann et al., 2002) is thought to be driven by mechanisms distinct from those driving diffuse-axonal degeneration and atrophy associated with disease progression (Ingle et al., 2003; Kutzelnigg et al., 2005). Relapsing-remitting (RRMS) presents with alternating clinical attacks and periods of stability that transition into secondary progressive MS (SPMS) with progressive deterioration. Approved disease modifying therapies (DMT) for MS target inflammatory processes and have demonstrated significant benefit in studies of RRMS (Yong et al., 2018), yet only recently have DMT been approved to give modest but significant benefits in progressive or primary progressive MS (PPMS). Specifically, the B-cell depleting antibody, ocrelizumab, has been shown to significantly reduce the confirmed disability progression over 12 weeks in ocrelizumab patients compared to those taking placebo (Montalban et al., 2017). Siponimod, a compound which traps B and T cells in the body’s immune organs has also shown evidence of slowing disability progression and has recently been approved for patients with active SPMS (Kappos et al., 2018).

Focal lesions are attributed to activated T cells that travel to the central nervous system (CNS) and trigger an inflammatory cascade as they encounter antigen (Bar-Or, 2008). CD4 and CD8 T cells may contribute directly and indirectly to disease but have been postulated to have both pathogenic and suppressive/regulatory roles over the disease course (Hauser et al., 2008; Racke, 2009; Johnson et al., 2010). Cells that induce EAE have a proinflammatory phenotype (Th1) producing interferon-γ (IFN-γ), tumor necrosis factor-α (TNF-α), and granulocyte macrophage colony stimulating factor (GM-CSF), in contrast to any of the immunosuppressive cytokines interleukin-4 (IL-4), IL-5, IL-9 or IL-10 (Gor et al., 2003; Sospedra and Martin, 2005). Additionally, the role of IL-17 producing T helper cells (Th17), as key drivers of pathogenic autoimmunity has been highlighted in EAE (Aranami and Yamamura, 2008). Yet, a balance seems to exist over the course of disease between effector T cells and regulatory T cells (Korn et al., 2007). Regulatory T cells (Treg) are capable of limiting immunity either by interacting directly with pathogenic cells, or by synthesis of immunosuppressive factors including IL-10 or transforming growth factor-β (TGF-β; Banham et al., 2006). Specifically, Treg cells expressing forkhead transcription factor FoxP3 are key cells capable of preventing disease in animal models of autoimmunity (Sakaguchi et al., 2008). Several factors in the local tissue environment can influence the phenotype of a T cell; inflammatory mediators or other cytokines, as well as the nature of the antigen presenting cell (APC), can greatly influence the properties of effector cells. Autoimmunity is typically reduced or even prevented if the generation of these pathogenic cytokine-producing cells deviates to a more immunosuppressive phenotype (Zamvil and Steinman, 1990; Segal and Shevach, 1998). As such, therapies which are able to reduce the development of autoreactive T cells, alter the pathogenic profile of these cells, or encourage the generation of regulatory T cells hold promise.

Mucosal tolerance describes the phenomenon of limiting immune responsiveness to antigens delivered *via* oral or nasal routes that would typically be considered non-pathogenic (i.e., food). Tolerance induction has been demonstrated *via* the intranasal route and is thought to involve active suppression as it can be transferred with adoptive transfer of splenocytes (Unger et al., 2003). In this regard, antigens delivered to the nasal mucosa spurs activation after draining into the cervical lymph nodes (CLN) and spleen which leads to tolerance; the internal jugular superficial cervical lymph nodes (SCLN) are also key in mediating tolerance (Wolvers et al., 1999; Li et al., 2011). Intranasal and oral delivery of antigen for tolerization has been effective in animal models of autoimmunity, atherosclerosis, transplant rejection, and allergy (Maron et al., 2002; George et al., 2004; Mayer and Shao, 2004; van Puijvelde et al., 2006; Broere et al., 2008; Klingenberg et al., 2010).

E-selectin is a cell surface adhesion molecule rapidly and specifically induced on activated endothelium which along with P-selectin, mediates tethering and rolling of immune cells *in vivo* and *in vitro* (Lawrence and Springer, 1991, 1993; Frenette et al., 1996). E-selectin has been implicated in processes of rolling and recruitment in EAE (Engelhardt et al., 1997; Doring et al., 2007), and soluble plasma levels have been associated with inflammatory activity in MS (Kuenz et al., 2005). Nasal instillation of E-selectin was previously shown to limit damage secondary to both ischemic and hemorrhagic strokes and to be protective in a vascular cognitive impairment model (Takeda et al., 2002, 2004; Wakita et al., 2008). E-selectin administration gave rise to a cell-mediated protection in models of stroke (Chen et al., 2003) as well as limiting atherosclerosis in susceptible *Apoe*^−/−^ animals (Li et al., 2011). This study was designed to test whether tolerization to human E-selectin *via* intranasal administration could alter the course and severity of disease in EAE as a model of MS.

## Materials and Methods

### Intranasal Administration of E-selectin

Experiments were approved and conducted in accordance with the Guide for the Care and Use of Laboratory Animal Resources (1996) and the Canadian Council on Animal Care guidelines, with approval by the NINDS Animal Care and Use and UBC Animal Care Committees. Intranasal instillations were as follows: (1) PBS (Biowhittaker, Walkersville, MD, USA); and (2) recombinant human (hu) or mouse (m) E-selectin (Supplementary Figure S1) synthesized with a Baculovirus expression system with lectin and epidermal growth factor domains (prepared and provided by Novavax Pharmaceuticals, Rockville, MD, USA). Instillations were carried out with the animals under brief anesthesia with 4% isoflurane using a 10 μl pipette tip and micropipettor.

**Figure 1:**
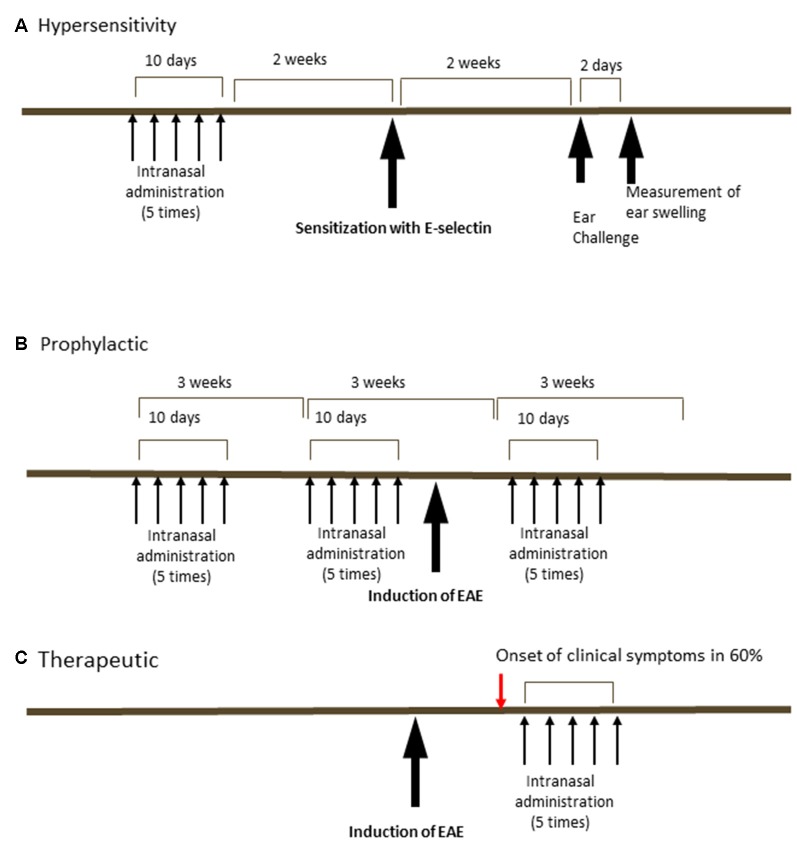
Schedules of intranasal delivery of E-selectin in delayed-type hypersensitivity (DTH) assays or prophylactic or therapeutic regimens in experimental autoimmune encephalomyelitis (EAE). **(A)** In a DTH reaction, animals received varying doses of ovalbumin or E-selectin in 10 μl PBS every other day for 10 days ending 2 weeks prior to sensitization with E-selectin. Animals were challenged in the ears 2 weeks later and swelling measured 48 h later. **(B)** In a prophylactic regimen, animals received E-selectin or ovalbumin over two periods prior to the induction of EAE and again 9 days after the induction of EAE. **(C)** In a therapeutic regimen, E-selectin was administered intranasally starting 2 days after the onset of clinical symptoms in EAE mice (red arrow, typically onset was day 14, with administration started at day 16 and delivered for 10 days).

The tolerization regimens changed over the course of the study (Figure 1) depending on whether it was administration of E-selectin to limit delayed-type hypersensitivity (DTH; Figure 1A), prophylactic administration of E-selectin prior to active EAE induction to prevent EAE (Figure 1B), or therapeutic administration of E-selectin after the onset of clinical symptoms (Figure 1C) PBS (10 μl), E-selectin or ovalbumin (Ova; varied doses in 10 μl) were instilled into each nostril every other day for 10 days (total of five administrations), designated as a single-course regimen. For EAE studies, a repetitive (booster) regimen was also applied, where the booster would be repeated at 3-week intervals.

### Delayed-Type Hypersensitivity Reactions (DTH)

DTH was assessed in C57BL/6 animals receiving a single-course tolerization regimen (five administrations every other day over a total of 10 days per regimen) as previously described (Li et al., 2011). Fourteen days later, the animals were immunized in four spots on the flank with 55 μg E-selectin/200 μl of 0.5mg/ml complete Freund’s adjuvant in PBS (Difco Laboratories, Detroit, MI, USA). Fourteen days later, ear thickness was measured with microcalipers and animals were subsequently re-challenged with 5 μg E-selectin/10 μl PBS injected subcutaneously into the ear. Ear thickness increase over baseline was measured 2 days later and presented as percentage increase and compared to increases following a PBS injection in the alternate ear.

### EAE Induction

For active disease, female 8–10-week-old C57BL6/J mice (Jackson Labs) were immunized with 200 μg myelin oligodendrocyte glycoprotein (MOG)_35–55_ (MEVGWYRSPFSRVVHLYRNGK; Stanford Pan Facility, Stanford, CA, USA; Quandt et al., 2012). For passive disease, SJL/J mice (Harlan Laboratories) were immunized with 75 μg PLP139–151 (HCLGKWLGHPDKF, Stanford Pan Facility) and draining LN cells enriched with PLP for 3 days in culture before the transfer of 1.5 million blasted cells to healthy recipients as described (Anderson et al., 2004). Animals were monitored by a blinded assessor daily for clinical signs through the onset of disease and through recovery (typically day 30–35) or longer in chronic studies to day 45 or 60 according to the following: 0–0.5 indicated no disease/distal limp tail, 1.0 limp tail, 2.0 mild weakness in one or 2.25 in two hindlimbs/slipping on cage insert bars, 2.5 moderate-severe weakness in one or in 2.75 two hind limbs/getting stuck on cage insert bars, 3.0 paralysis in one or 3.5 both hind limbs, 4.0 hind limb paralysis plus weakness in one or in 4.5 both forelimbs, and five for moribund animals.

### Isolation of Immune Populations for Transfer or T Cell Proliferation and Cytokine Assays

The spleens or SCLN were aseptically removed from mice following CO_2_ inhalation and euthanasia and transferred to cold sterile PBS and pressed through a sterile 40 μm filter to prepare a single-cell suspension. For transfer, cells were resuspended in a sterile PBS buffer and incubated with antibodies specific for non-T cell populations to prepare CD4+ T cell-enriched populations by negative selection (Miltenyi Biotec, Gladbach, Germany); a small sample was kept for characterization by flow cytometry which showed CD4+ T cell purities ranged from 93% to 96% in each experiment. Five million cells from tolerized animals were immediately transferred retroorbitally to recipient animals which had been induced for passive EAE as above. For analyses of immune responses or priming, 2–4 × 10^5^ spleen or LN cells in serum-free X-vivo 15 media (BioWhittaker, Walkersville, MD, USA) were incubated with varying doses of E-selectin, control culture media, or concanavalin A (Con A; final concentration 2 μg/ml, Sigma, MO, USA) in 96-well round bottom plates for 48 h at 37°C when tissue culture supernatants were harvested. Thymidine (1 mCi of [^3^H]-thymidine) was added for 16 h prior to harvesting the cells at 72 h and measurement of incorporated radioactivity. Cytokine analysis was performed using anti-mouse IFN-γ IL-10-, IL-17 or TGF-β-specific DuoSet kits from R&D Systems (Minneapolis, MN, USA) per protocol directions.

### Serum Analyses

Blood was collected from the tail vein *via* sterile scalpel from mice anesthetized under 4% isoflurane. Samples sat at room temperature for 30 min in a Serum Gel Z/1.1 tube (Sarstedt, Numbrech, Germany) prior to processing per manufacturer’s recommendations and frozen at −80°C until assay. Serum E-selectin was measured using a mouse E-selectin-specific Duoset kit from R&D Systems as above and MOG_35–55_ specific IgG1 and IgG2a/c antibodies were detected as previously described (Neil et al., 2017).

### Immunohistochemistry of EAE Tissues

Mice were deeply anesthetized with a ketamine/xylazine cocktail prior to transcardial perfusion with PBS followed by 10% buffered formalin and processed for immunohistochemistry (IHC; Quandt et al., 2012). Immunofluorescence with antibodies to SMI-32 (to hypophosphorylated neurofilament, Biolegend, San Diego, CA, USA) and myelin basic protein (MBP, Santa Cruz Biotechnology, Dallas, TX, USA) were used to assess infiltration, axonal damage as well as demyelination in the spinal cord. Tissue sections were examined with a Zeiss Axio Vert 200 Inverted Fluorescence Microscope (Oberkochen, DE, Germany), equipped with an Axiocam 506 monochrome camera at 20× with matched light intensity and exposure times across the entire experimental set. Analysis was performed with Zeiss software Zen (Version 2.3). Immunostained sections from each EAE mouse spinal cord were analyzed (*n* = 5 per treatment, three levels per mouse corresponding to the T8/T9, T12, and L4/5 levels of the spinal cord for a total of 15 levels per treatment group examined). The sections were assessed for the degree of inflammatory infiltration by outlining and pooling the area of all infiltrates per section and calculating the percentage of white matter (WM) infiltrated by immune cells by taking this as a fraction of the WM area in that section. SMI32-positive regions of interest typically resembling spheroids with a staining intensity above the threshold set by averaging regions of WM in healthy animals were used to report the number of SMI32+ spheroids per square millimeter of WM.

### Statistical Analysis

GraphPad Prism (version 7, La Jolla, CA, USA) was used for all analyses. A repeated-measures one-way analysis of variance (ANOVA) was used for normally distributed data or a repeated measures ANOVA on ranks (Friedman test) was performed when comparing multiple treatments. In EAE comparisons of multiple doses over time, a two-way ANOVA on ranks was performed followed by a Tukey multiple comparisons test vs. PBS-treated controls. Values represent mean ± standard deviation unless otherwise indicated. A Mann-Whitney comparisons test was performed in comparisons of EAE to healthy or sham/CFA immunized mice and significance reported at *p* < 0.05.

## Results

### Intranasal Administration of E-selectin Specifically Limits Inflammation Secondary to Immunization With E-selectin or EAE Induction With MOG

**Figure 2:**
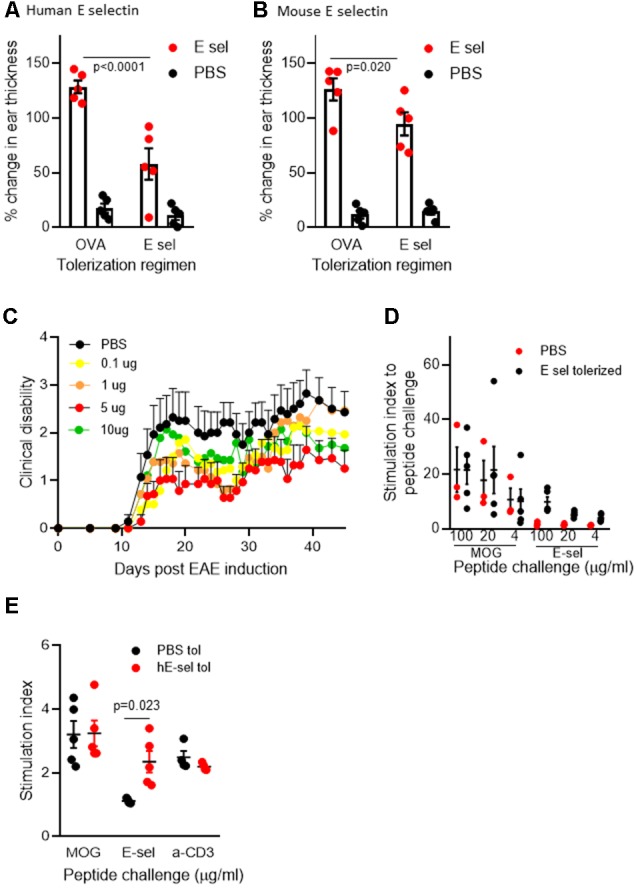
Intranasal E-selectin limits DTH responses and limits clinical disability in EAE. **(A)** Percentage change in ear thickness in animals sensitized to huE-selectin after intranasal administration of 5 μg E-selectin or Ova and challenged with PBS or huE-selectin in opposite ears. **(B)** Percentage change in ear thickness in animals sensitized to mE-selectin after intranasal delivery of mE-selectin or Ova. No differences in ear thickness were observed between ears receiving PBS challenge following either regimen. *n* = 5 mice per treatment group, bars represent the SEM. Mann-Whitney *T*-test comparing Ova to E-selectin tolerization. **(C)** huE-selectin was administered per the regimen in Figure 1B spanning the active induction of chronic EAE. 0.1 up to 10 μg of huE-selectin were administered and results represent the mean ± SEM of the animals over time. **(D)** Splenocytes isolated from animals at D.45 post-EAE induction were challenged *ex vivo* with different doses of MOG_35–55_ or huE-selectin for 72 h to examine the proliferation relative to untreated samples (stimulation index or SI). *n* = 3–4 animals per group, dots and error bars represent the average SI of responses from PBS (black) or huE-selectin tolerized (red) animals ± SD. Responses to huE-selectin in PBS tolerized animals were negligible. **(E)** Cells isolated from the draining axillary, brachial and inguinal lymph node (LN) 12 days after EAE induction were tested for proliferative responses to MOG_35–55_ (20 μg/ml), E-selectin (10 μg/ml) and anti-CD3 combined with anti-CD28 (*n* = 4–5 mice per group, stimulation index (SI) is proliferation relative to untreated samples from PBS—black or E-selectin-red-tolerized animals ± SEM).

In C57BL/6J mice, animals sensitized and later undergoing recall to either human or mouse E-selectin *in vivo* in a DTH assay (Figure 1A) showed similar responses: the average increase of ear thickness in mice sensitized with hE-selectin and recalled later with hE-selectin was 0.331 ± 0.02 mm and was similar to mice sensitized with msE-selectin and later challenged with mE-selectin at 0.324 ± 0.06 mm. In animals sensitized to huE-selectin, intranasal administration of huE-selectin limited swelling to 57.9 ± 4.2% compared to 128.5 ± 3.8% observed in animals receiving Ova intranasally (Figure 2A, 5 μg dose; *p* < 0.0001). Similarly, intranasal msE-selectin limited swelling in msE-selectin-sensitized mice although to a lesser degree (94.7 ± 5.4% vs. 126.2 ± 7.4% in Ova-tolerized animals Figure 2B; *p* = 0.02). A lower dose of 1 μg still significantly reduced hE-selectin responses whereas 1 μg of mouse E-selectin was not effective (data not shown). Notably, human and mouse E-selectin proteins share 81% amino acid similarity.

**Figure 3:**
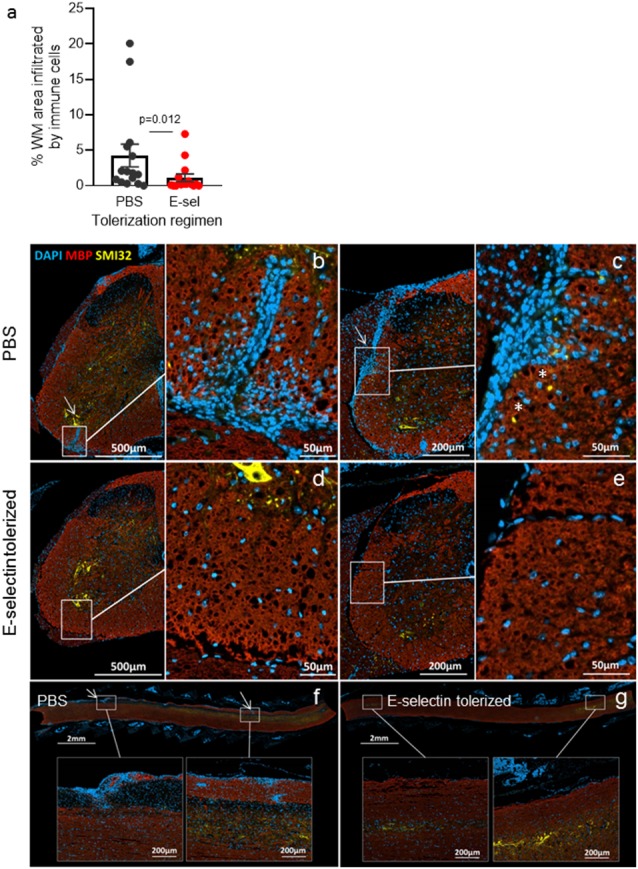
Immune infiltrates are significantly reduced in E-selectin tolerized mice. **(A)** Animals receiving PBS (black) or huE-selectin (red) for one round prior to EAE induction were euthanized after the onset of disease (D.12) to examine the percentage of white matter (WM) infiltration by immune cells in the spinal cord. *N* = 5 mice analyzed at three levels per treatment group; bars represent the mean and error bars the SEM of 15 levels per treatment group. Representative images from mice in panel **(A)** are shown. Images and insets were taken from different levels of PBS tolerized **(B,C)** and E-selectin-tolerized **(D,E)** animals. Arrows highlight regions of extensive immune infiltrate from the meninges that extend into the parenchyma that were typically only present in animals receiving PBS intranasally. MBP (red) loss was detected in PBS-tolerized animals in heavily infiltrated areas, as were SMI-32+ ovoids reflecting axonal damage (*). Longitudinal sections also demonstrate the extensive inflammatory infiltrates detected in animals receiving intranasal PBS **(F)** vs. E-selectin **(G)**.

We next administered hE-selectin intranasally to test its potential to limit autoimmunity and clinical disease in the actively induced MOG_35–55_ chronic model of EAE. Animals received 0.1–10 μg of hE-selectin in three sessions which precede and span the induction of EAE as in Figure 1B. Compared to intranasal PBS, 5 μg of hE-selectin reduced clinical disability in animals to the greatest degree, although 0.1 and 10 μg doses were also effective (Figure 2C). Intranasal administration of 5 μg hE-selectin significantly reduced the cumulative disease burden over the study (37.1 ± 9.4 vs. 70.3 ± 13.7 in PBS, *p* = 0.05) and the average disease severity (1.16 ± 0.27 vs. 2.141 ± 0.42 in PBS, *p* = 0.05). Comparisons of the disease score over time showed that the means of each treatment were significantly different to PBS (PBS vs. 0.1 μg, *p* < 0.0001, PBS vs. 1 μg, *p* = 0.0002, PBS vs. 5 μg, *p* < 0.0001, PBS vs. 10 μg, *p* = 0.0007). We postulated that intranasal administration of E-selectin may limit the magnitude of a response to MOG in these animals, and examined spleens from animals culled at day 45 and tested recall *ex vivo* to both MOG and hE-selectin. Notably, stimulation indices to MOG were not significantly different between the two groups (Figure 2D). Furthermore, hE-selectin-specific responses could only be detected in populations derived from E-selectin tolerized animals. Notably, we have found that the isotype of antibody responses to MOG_35–55_ can be a sensitive means of detecting shifts in Th1 or Th2 profiles (Neil et al., 2017) and found that IgG1 and IgG2a antibody responses to MOG_35–55_ were comparable in PBS and hE-selectin serum from mice examined at day 45 tolerized with the regimen in Figure 2C (data not shown). To determine whether or not E-selectin tolerization interfered with the initial priming of encephalitogenic T cells, draining inguinal, axillary and brachial LN populations were isolated from animals receiving one round of intranasal PBS or E-selectin and actively immunized to induce EAE. Animals were culled 12 days after immunization and tested for proliferative responses to MOG, hE-selectin, and anti-CD3 as well cytokine production over 48–72 h. Proliferation responses measured by stimulation indices to MOG_35–55_ and anti-CD3 were similar between PBS and E-selectin tolerized animals (Figure 2E); however, proliferation in response to E-selectin was only detected when E-selectin had previously been administered (*p* = 0.023). Both IL-17 and IFN-γ production, in response to 20 μg/ml of MOG_35–55_, was similar in cells derived from E-selectin tolerized LN as from those receiving PBS (596 ± 84.3 pg/ml vs. 654.8 ± 189 pg/ml and 426 ± 64.8 vs. 502 ± 72.1 pg/ml respectively). Polyclonal T cell activation with anti-CD3 and CD28 also yielded similar IL-17 and IFN-γ amounts from the two different treatment groups (*n* = 5 mice per treatment group). No IL-17 or IFN-γ was detected in either group in response to E-selectin and production of TGF-β was negligible across all tolerization and stimulation groups (data not shown). IL-10 levels were near the lower limit of detection (0–16 pg/ml) and was not significantly different between PBS and E-selectin tolerized populations no matter the stimulus. At day 12, animals receiving PBS scored at 0, 2, 1.5, 1 and 2.5; animals receiving huE-selectin scored at 0, 0, 0, 0 and 2.75. Examination of three levels of spinal cord showed that animals which received intranasal E-selectin had inflammatory infiltrates in less than 1/3 of the tissue observed in PBS tolerized mice (Figure 3A; 1.12% ± 0.5 of the tissues vs. 4.27% ± 1.6 of the tissue, *p* = 0.012). No significant differences in the accumulation of SMI-32+ spheroids as a marker of axonal damage were observed at this time point (Figures 3B–G; data not shown).

### Therapeutic Potential of E-selectin Tolerization and Immune Responsiveness

**Figure 4:**
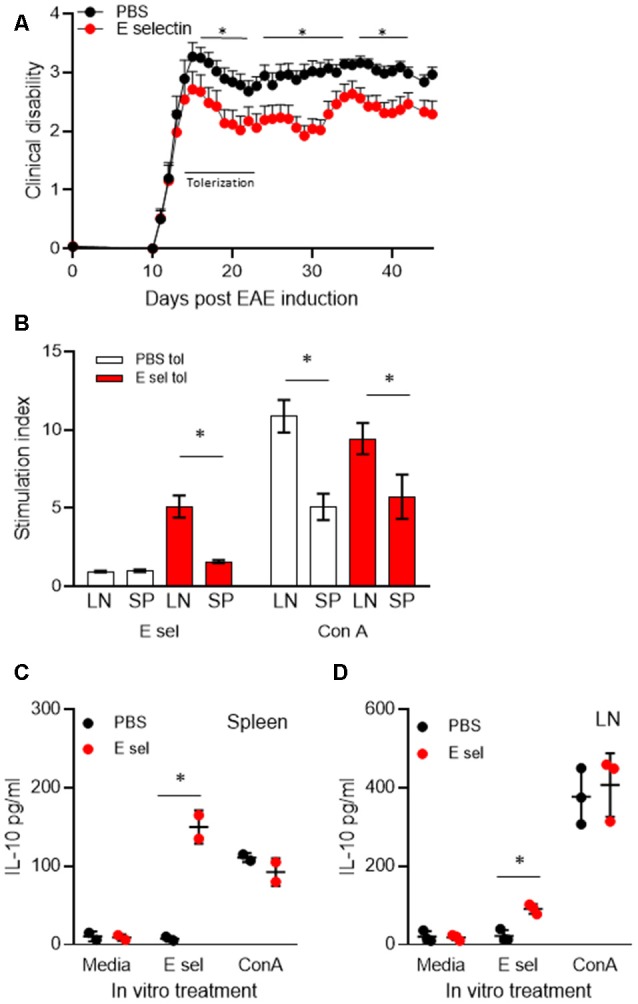
Administration of E-selectin treats disability in chronic EAE and generates E-selectin-specific populations in immune organs. **(A)** Mice were immunized with myelin oligodendrocyte glycoprotein (MOG)_35–55_ to actively induce EAE. Once more than half the animals exhibited a limp tail (day 13) huE-selectin tolerization was initiated every other day for 10 days. Animals were followed to day 45. Average disease scores were significantly reduced over the treatment study, *p < 0.05 *t*-tests vs. PBS-treated mice indicated those days which were significant. *n* = 13 and 14 for the PBS and E-selectin groups respectively. One of three representative experiments is shown. **(B)** Superficial LN and splenic populations were isolated from the mice in **(A)** at day 45 and challenged *ex vivo* with E-selectin or Con A to assess the response to antigen. Proliferative responses over 72 h to 20 μg/ml huE-selectin (left panel) were only observed in LN and spleen populations which had received E-selectin intranasally (red bars). Data represents spleen and cervical lymph nodes (CLN) from three mice per treatment group, average ± SD. **(C,D)** Supernatants from the splenic and LN populations isolated from animals undergoing E-selectin tolerization were examined for IL-10 production, PBS or huE-selectin. Spleens from *n* = 2 or from LN *n* = 3 mice were assayed per regimen treatment group, bars represent the average produced from duplicate or triplicate wells, error bars represent the SD. *p* < 0.05.

Administration of huE-selectin after the onset of disease symptoms was tested for the potential to treat disease in chronic EAE. Administration of huE-selectin after the onset of disease (starting on day 13 for 10 days as in Figures 1C, 4A) reduced the average disease score over the study period (2.60 ± 0.13 vs. 2.12 ± 0.09, *p* = 0.0006). To characterize the specificity of the immune response to E-selectin, superficial cervical LN and splenic populations were examined from mice at the end of the study period (d.45 post-immunization) for recall to E-selectin and compared to the T cell mitogen Con A (Figure 4B). E-selectin-specific responses were only detected in animals that had been tolerized to E-selectin. Likely consistent with fewer T cells in the spleen than LN, the splenic responses to E-selectin were less than those observed in the LN (1.58 ± 0.18 vs. 5.72 ± 2.45, *p* = 0.03). Responses to Con A were similarly lower in spleens than LN in both groups of tolerized animals, and no differences were observed between the proliferative responses of cells derived from the PBS vs. E-selectin tolerized animals. Supernatants from spleen cells and LN cells isolated from PBS or E-selectin tolerized animals were tested for IL-10 production; IL-10 production in response to E-selectin was only detected in animals that had received E-selectin (Figures 4C,D) where it was significantly greater (*p* < 0.05) than that observed in PBS-tolerized animals. Notably, IL-10 production in response to Con A was similar from both PBS and E-selectin tolerized animals.

**Figure 5:**
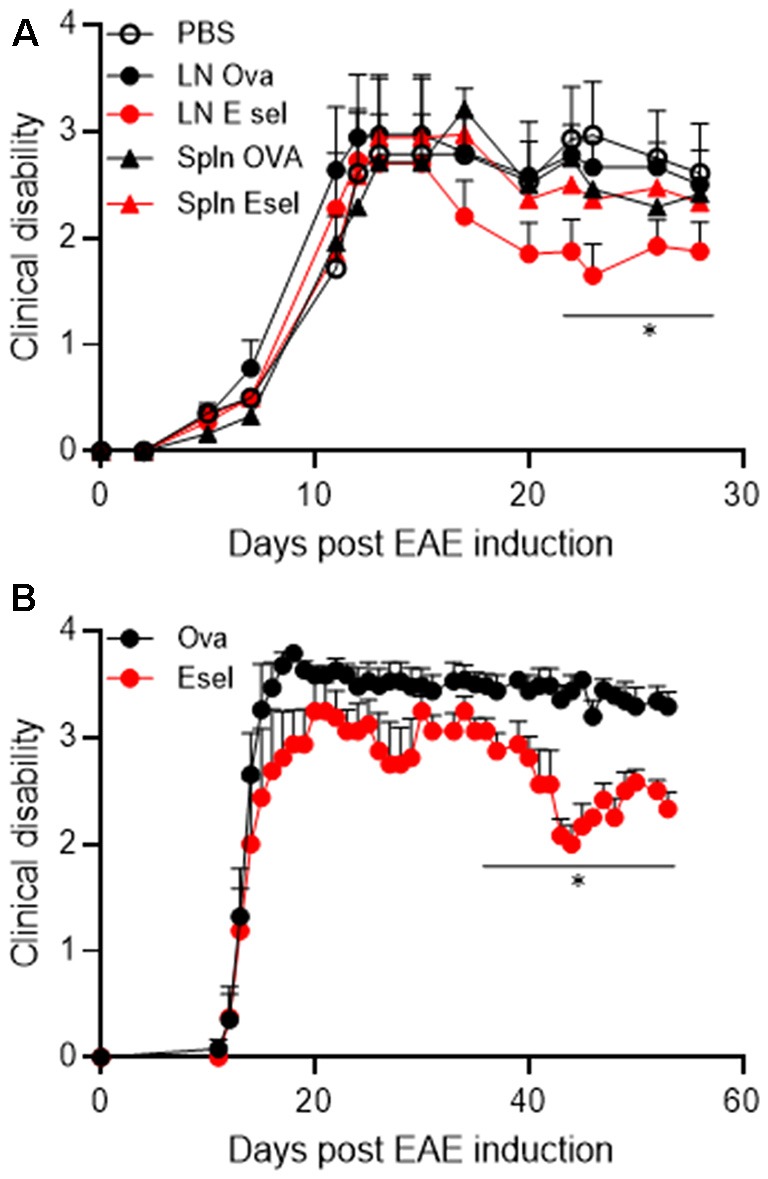
**(A)** Superficial cervical LN populations protect against passive EAE in SJL mice. SJL animals received 1.5 million blasted PLP_139–151_-specific T cells intraperitoneally to induce disease. Purified CD4+ T cells isolated from Ova (black) or huE-selectin (red) tolerized animals (two rounds) were transferred 5 days later. Clinical scores were assessed daily until day 30. Points represent the average clinical score of *n* = 8–11 mice per group, errors bars the SEM. *p < 0.05 compared to animals receiving PBS. **(B)** A replicate of the experiment in panel **(A)** where CLN cells from OVA or huE-selectin tolerized mice were transferred after CD4+ T cell purification. Points represent the average clinical score of *n* = 5 and 6 mice per group, errors bars the SEM. *p < 0.05 compared to Ova-tolerized animals.

Our group had previously shown that transfer of splenic cells from E-selectin tolerized cells was able to confer protection in experimental models of disease and we sought to test the same in EAE (Figure 5). To focus on CD4+ T effector populations, we purified CD4+ T cells from the superficial cervical LN or spleens of animals that had undergone two rounds of intranasal administration (per Figure 1) with PBS, OVA or E-selectin and transferred them into animals induced passively for PLP_139-151_ EAE.

Animals receiving splenic cells from huE-selectin or Ova-tolerized animals or LN cells from Ova-tolerized animals all had similar average disease severities over the study period to PBS-tolerized animals (1.87 ± 0.30, 1.84 ± 0.31, 2.05 ± 0.31 and 1.95 ± 0.32); only animals receiving LN cells from E-selectin tolerized mice were different at 1.611 ± 0.25 (*p* < 0.05 over the disease course; Figure 5A). In a replicate of the study over 60 days, LN cells from huE-selectin tolerized animals had a significantly lower disease score over the study than cells from Ova tolerized animals (3.16 ± 0.15 vs. 2.51 ± 0.13, *p* < 0.0001; Figure 5B). LN populations were assessed by flow cytometry prior to transfer in each experiment, with 94.3%–96.5% CD4 T cell purity and within that population 10.3%–12.6% FoxP_3_+/CD25+ cells in the Ova vs. huE-selectin transferred populations, showing no significant difference in T reg numbers between the two transferred populations.

## Discussion

The microvasculature of the brain exhibits specialized tight junctions, low levels of vesicular trafficking and limited cell adhesion molecule (CAM) expression that otherwise enables immune cell movement out of the vessel (Reese and Karnovsky, 1967; Risau et al., 1998). Therapies which limit endothelial cell activation or immune cell recruitment across the blood brain barrier (BBB) offer promise in MS. Indeed, natalizumab, an antibody to α4 integrin the ligand for vascular CAM-1 (VCAM-1), was extremely effective in treating EAE (Zhang et al., 2003) and went on to show the ability to reduce relapses in MS patients by 68% compared to placebo, which far exceeded results for approved MS therapies at that time (Polman et al., 2006). However, this, and more recently approved efficacious therapies, have an increased risk profile, where depleting immune cell subsets or reducing immune surveillance with monoclonal antibodies were associated in rare instances with multifocal leukoencephalopathy (PML) as a rare yet serious adverse event (Piehl et al., 2011; Yong et al., 2018).

The current study uses a novel approach enabling expression of E-selectin under inflammatory conditions to localize and perpetuate an immunoregulatory response. Intranasal administration of E-selectin was a driver of specific immune responses in both the SCLN and spleen which were associated with the expression of the immunomodulatory cytokine IL-10 (Li et al., 2011), as previously described.

In our studies, we compared the efficacy of purified CD4+ T cells from SCLN to splenic populations and found E-selectin-specific cells derived from SCLN after two rounds of priming had efficacy. Many studies have reported intranasal antigen administration as a means to regulate CD4+ T cell function and have linked IL-10 to subsequent immune tolerance (Hoyne et al., 1996; Burkhart et al., 1999; Laliotou and Dick, 1999). In an experimental autoimmune uveitis model, authors showed a transfer of tolerance with a regulatory population of spleen cells (Burkhart et al., 1999; Laliotou and Dick, 1999). Studies have implicated APCs as key mediators of tolerance, particularly those in the regional drainage lymph nodes; a single intranasal administration was rapidly followed by T cell proliferation secondary to signaling networks in both SCLN and spleen (Wolvers et al., 1999). It may be that the multiplicity and boosting of these administrations in our model contributed to the increased efficacy observed upon transfer of SCLN-derived populations, which was also apparent in the increased proliferative responses observed when comparing E-selectin responses between the two immune organs.

The administration of E-selectin repeatedly, as with any antigen, has the capacity to generate both T and B cell responses specific to E-selectin. Although we previously showed that antibodies specific for E-selectin are generated using this approach, we showed that they are indeed of the IgG1 isotype, and are therefore a more immunsuppressive than inflammatory phenotype. This, combined with previous observations from other groups which show that neutralization of E-selectin with antibodies does not reduce the severity of EAE, suggests that the primary mechanisms of action here are not attributable to antibodies generated from T cell-aided B cell responses. It is well known that administration of antigens through mucosal routes rapidly generates T cell populations, immunomodulatory in nature, which, through their production of cytokines such as IL-10, can limit inflammation. Numerous reports have outlined the relative success of antigen-specific tolerance to limit EAE based on the administration of the candidate autoantigen driving the disease, although discussion surrounds the safety and also reliability of mucosal administration of antigen to suppress ongoing active disease (Burkhart et al., 1999). To test the efficacy of E-selectin administration in the most robust manner, we tested the approach prophylactically and therapeutically in active and passive models of chronic and relapsing-remitting MS, to overcome previous concerns that only in more of the milder relapsing disease settings would tolerance approaches have success (Liu and Wraith, 1995; Bai et al., 1997; Thurau et al., 1997).

The very nature of the tolerizing antigen in our approach may be the key to the success here; rather than having to energize or deviate the full weight of the immune response to the candidate autoantigen, E-selectin tolerization and the potential of E-selectin specific cells to act at the specific site of inflammatory responses and damage may significantly augment its potential to limit disease, without the risk of overt immunosuppression. Furthermore, in the case of MS where the definitive putative autoantigen remains elusive, the specific autoantigen need not be identified for this approach to yield benefit. Here, we target immunosuppressive cell populations towards the activated microvasculature and/or local immune organs; the specific sites remain to be identified. Future studies will be important to establish the site where these cells act to afford protection. There is indeed the possibility that the human E-selectin gives a more robust response as seen in the DTH because it differs from the mouse protein in sequence. It is also possible that any differences observed in the glycosylation patterns of otherwise homologous regions of the protein may be enhancing immunogenicity following intranasal administration. It is possible that E-selectin from another species from human may be more potent in humans, but may increase the potential for autoimmunity if high avidity cells specific for a non-human protein that have not been depleted through tolerance to self (human) antigen are indeed activated. Because self into self in our model was not a clear driver of autoimmunity, yet showed efficacy albeit lower than that observed with that from a different species, we believe a homologous system is a safe approach to consider in driving antigen-specific tolerance to E-selectin.

Despite E-selectin upregulation in association with activation of the vasculature in a variety of CNS insults or inflammatory settings and their models, a specific requirement for E-selectin in EAE development has been discounted. Intravital microscopy experiments showed that E-selectin is localized to regions of immune cell rolling and interactions with the superficial CNS microvasculature (Kerfoot et al., 2006); moreover, E- and P-selectin mediate early cerebrovascular T cell/endothelial interactions in EAE, and P-selectin glycoprotein ligand-1 (PSGL-1, one ligand for E- and P-selectin) is a primary mediator of E- and P-selectin-mediated T cell rolling in the spinal cord (Sathiyanadan et al., 2014). In contrast, studies have: (a) failed to localize E-selectin to parenchymal vessels over the course of EAE development; and (b) have shown that neutralization of E-selectin with antibodies or E-selectin knockout models show no differences in clinical disease or infiltrate composition compared to their wildtype counterparts (Engelhardt et al., 1997). In this regard, one concludes such rolling of T cells is not required for the initiation of EAE. However, elegant studies in relapsing and remitting remodels of disease have shown an important role for neo-epitope generation in potentiating disease through antigen presentation and the potentiation of immune responses to newly generated antigens within the CNS as proposed in antigenic “spread” (McMahon et al., 2005). Notably, relapse in chronic-relapsing EAE is less severe where CNS-draining lymph nodes have been removed (van Zwam et al., 2009). E-selectin draining to these lymph nodes during EAE or MS may be the driver for IL-10 responses that limit immune responses here, and foreseeably further potentiation. Future experiments will build on this initial study, and look to further characterize E-selectin-specific populations and their potential to limit inflammation both *in vitro* and *in vivo*.

*In vitro* models and the postulated pathogenesis of MS suggest that the BBB is not restrictive to trafficking of activated lymphocytes (Goverman, 2009). In our model, where T cells specific for E-selectin are generated and repeatedly challenged, this may lead to E-selectin specific cells that can gain entry to the CNS, even when the BBB has not been compromised. If these cells access the CNS and can go on through the cerebrospinal fluid to the recently characterized CNS meningeal lymphatics which then drain to cervical lymph nodes (Louveau et al., 2015), exposure here to E-selectin may drive their production of immunomodulatory factors such as IL-10 which may help to limit antigenic spread and the potentiation of immune responses that are more likely to start within the CNS. Additional studies are required to titer as well to localize E-selectin-specific T cells in our model over the course of EAE to better understand their full potential to actively and passively protect against and treat established disease in our models.

While rapid expression of E-selectin at the CNS microvasculature surface at a specified point in the disease process may be the most robust signal of inflammation in acute settings such as stroke, perhaps equally as important is the shedding of E-selectin. While not characterized over the course of disease, we did detect mouse E-selectin in the serum of animals undergoing acute attacks of EAE (ranging from 250 to 300 pg/ml); notably, these levels were highest in animals undergoing EAE that had been tolerized with Ova; levels were significantly lower in animals that had been tolerized with human E-selectin suggesting that the relative level of serum E-selectin may be linked to the severity of disease and/or inflammation. In our therapeutic studies of E-selectin administration after the onset of disease, we saw no difference in serum E-selectin levels between animals receiving PBS or E-selectin 1 month (588.0 ± 15.1 vs. 566.6 ± 11.9 ng/ml) or 2 months (310.5 ± 12.1 vs. 366.5 ± 13.3 ng/ml) after disease induction, although soluble E-selectin had clearly dropped by almost half over that time. Notably, sE-selectin is markedly increased in RRMS patients during an exacerbation or those with chronic progressive MS in comparison to controls (Tsukada et al., 1995). Notably, MS was distinct from HTLV-1 associated myelopathy (HAM) in this regard where levels of sE-selectin were similar between patients with HAM and controls (Tsujino et al., 1998). Authors also frequently found sE-selectin in the CSF during exacerbations in RRMS, supporting a role for E-selectin–mediated responses to endothelial cell activation or damage during an exacerbation (Droogan et al., 1996). In contrast, others found CSF and serum sE-selectin levels were similar between patients in relapse and those with other inflammatory (IND) and non-inflammatory neurological disease (NIND). Another report sought to determine whether concentrations of sE-selectin correlated with gadolinium-DPTA enhancement (a marker of enhanced BBB permeability) on MRI in patients with MS. Authors found levels of sE-selectin were significantly increased in PPMS patients compared to the near control levels of sE-selectin detected in RRMS and SPMS patients. Interestingly, 5 of the 10 primary progressive patients with high levels of sE-selectin showed very low activity on MRI scans. While there was no correlation between sE-selectin concentrations and contrast-enhancing lesions on MRI, a close correlation was observed between TNF-α and sE-selectin (Giovannoni et al., 1996). Other groups have documented the same high levels of serum sE-selectin in PPMS compared with RRMS and SPMS (McDonnell et al., 1999) and provide evidence for significant immunological heterogeneity in MS related to immune/endothelial cell interactions in different types of MS. It also goes against the dogma of lesser inflammatory involvement that has previously characterized PPMS. This unique association between serum E-selectin and PPMS may indeed highlight the importance of advancing the therapeutic consideration of E-selectin tolerization to limit neurodegeneration in MS, with relevance to particularly acute and active processes such as those implicated in PPMS.

## Data Availability

All datasets generated for this study are included in the manuscript and/or the Supplementary Files.

## Ethics Statement

### Animal Subjects

All experiments were carried out in compliance with the Guide for the Care and Use of Laboratory Animal Resources (1996), the Canadian Council on Animal Care guidelines and approved by the NINDS Animal Care and Use and UBC Animal Care Committees.

## Author Contributions

JQ performed EAE induction, scoring, data collection, analyses, immunoassays, cell culture, tissue isolation and preparation, prepared the first and subsequent versions of the manuscript, formulated the research plan and series of experiments with input from JH; and oversaw the experimental approach and design, reviewed data and analyses and prepared the manuscript including revising the manuscript critically for technical and scientific accuracy with JH. PB and EK performed immunohistochemistry and analyses of that data. All authors have reviewed the manuscript in entirety and are in agreement with the content of this work.

## Conflict of Interest Statement

The authors declare that the research was conducted in the absence of any commercial or financial relationships that could be construed as a potential conflict of interest.
