# Supplementary figures and images for: Mucosal Administration of E-selectin Limits Disability in Models of Multiple Sclerosis
Source: Front Mol Neurosci. 2019 Aug 27;12:190. doi: 10.3389/fnmol.2019.00190 (PMC6718462; doi:10.3389/fnmol.2019.00190)

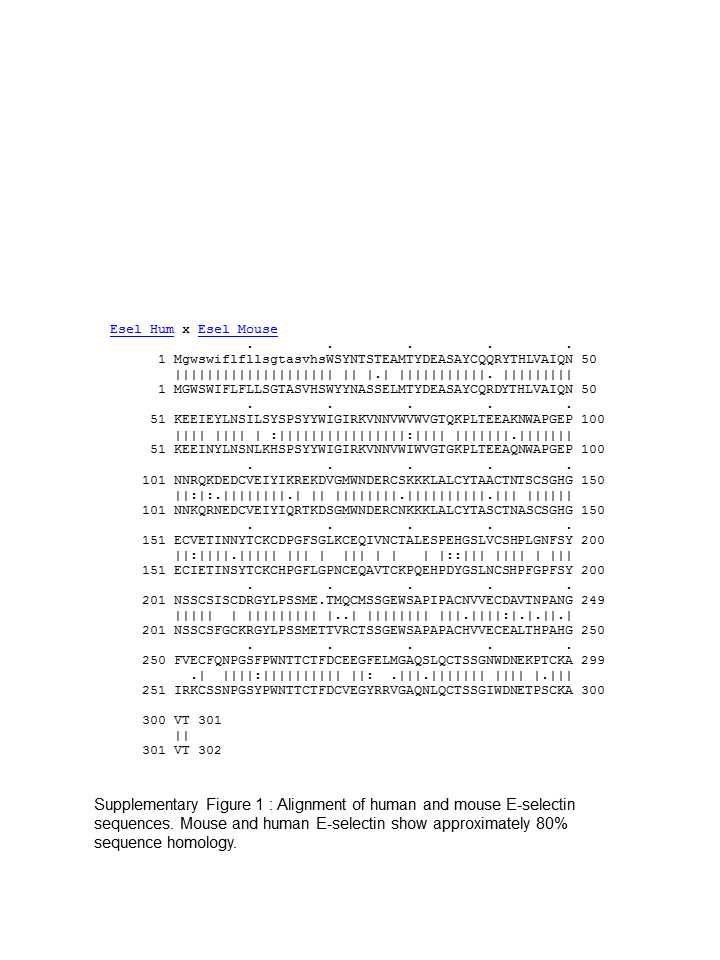

Supplement: Supplementary file 1 [file Image_1.TIF]
